# Clinical Outcomes of “U” Route Transforaminal Percutaneous Endoscopic Lumbar Discectomy in Chronic Pain Patients with Lumbar Spinal Stenosis Combined with Disc Herniation

**DOI:** 10.1155/2021/6657463

**Published:** 2021-01-19

**Authors:** Binbin Wu, Xinyi Tian, Ce Shi, Chenchen Jiang, Jing Zhang, Gonghao Zhan, Danli Xie

**Affiliations:** ^1^Department of Pain Medicine, The Second Affiliated Hospital and Yuying Children's Hospital of Wenzhou Medical University, Wenzhou, China; ^2^Department of Orthopedics, The Affiliated Suqian Hospital of Xuzhou Medical University, Suqian, China; ^3^Department of Clinical Research Center, The Second Affiliated Hospital and Yuying Children's Hospital of Wenzhou Medical University, Wenzhou, China; ^4^China-USA Neuroimaging Research Institute, The Second Affiliated Hospital and Yuying Children's Hospital of Wenzhou Medical University, Wenzhou, China; ^5^Department of Microbiology and Immunology, School of Laboratory Medicine and Life Science, Wenzhou Medical University, Wenzhou 325035, Zhejiang, China; ^6^Key Laboratory of Laboratory Medicine, Ministry of Education of China, Wenzhou 325035, Zhejiang, China; ^7^Wenzhou Key Laboratory of Sanitary Microbiology, Wenzhou 325035, Zhejiang, China

## Abstract

**Introduction:**

“U” route transforaminal percutaneous endoscopic lumbar discectomy (PELD) was introduced for lumbar spinal stenosis (LSS) combined with disc herniation (DH) treatment. This study aims to explore the efficacy and safety of “U” route PELD on chronic pain patients with LSS combined with DH.

**Methods:**

Degenerative LSS combined with DH patients who underwent “U” route PELD were reexamined, and 80 patients were recruited and followed up for 2 years. The other 80 healthy individuals who were age- and sex-matched to the patients without chronic pain were enrolled as healthy controls. Minimum dura sac cross-sectional area (mDCSA) by MRI, Visual Analog Scale (VAS), Oswestry Disability Index (ODI), and modified MacNab outcomes were assessed. Emotional evaluation of pain catastrophizing and depression was documented with Pain Catastrophizing Scale (PCS) and Beck Depression Inventory (BDI), respectively, for patients before and after surgery and healthy individuals.

**Results:**

All patients were of the age range from 47 to 85 years, with an average of 59.5 ± 9.76 years. Symptoms duration was 114.6 ± 22.77 months, operation time was 87.7 ± 25.20 minutes, and the average hospital stay was 5.8 ± 2.81 days. Four patients quit, and hence, a total of 76 patients completed the follow-up. The results indicated that mDCSA was improved significantly after operation (*p* < 0.001), either low back and leg VAS or ODI decreased over time (*p* < 0.001), and the excellent-to-good rate was improved from 88.75% to 93.42% during postoperative 2 years (*p* < 0.05). Complications of dural tear, nerve root, or dysesthesia were reported in 5 patients, and all recovered after conservative therapy. The scores of pain catastrophizing were reduced after operation (*p* < 0.001), but no significance of BDI was found between patients and healthy controls (*p* > 0.05).

**Conclusions:**

The “U” route PELD seems an alternative to LSS combined with DH treatment, which might reach a better decompression and effectively improve chronic pain conditions. Still, the complications were potential and required further consideration.

## 1. Introduction

Chronic pain is defined as the pain that persists beyond normal healing time. It usually lasts for more than 12 weeks [1]. Lumbar spinal stenosis (LSS) and disc herniation (DH) are two of the most common etiologies in chronic pain patients. The major causative factors of LSS include hypertrophied faceted joints, osteophyte formation, and ligamentum flavum (LF) hypertrophy. There are a large part of LSS patients with disc protrusion, resulting in spinal canal narrowing and dural sac constriction [2, 3]. The mechanical compression on the nerve root, cauda equina, and/or vascular structure leads to symptoms including low back pain, intermittent neurogenic claudication and radiculopathy in lower extremities, and sensory and motor disturbances [4, 5]. The treatment aims to alleviate symptoms and improve life quality; however, surgery should be considered after conservative treatment is gone through and failed [2].

Traditionally, the decompression plus fusion surgery is regarded as the standard procedure for LSS treatment [6]. During the last decades, LSS can be treated via a unilateral approach to achieve bilateral decompression and spinal canal enlargement with microendoscopic discectomy (MED) [7]; however, the complications of MED, delayed postoperative recovery, and reduced satisfaction rate were reported [8, 9]. As a further development of minimally invasive spinal surgery, transforaminal endoscopic spine system (TESSYS) procedure was introduced [10] and has been demonstrated feasible and effective for lumbar disc herniation (LDH) treatment [11, 12]. Upon the improvement of PELD procedure performance and development of surgical instruments, the indications of PELD are being explored. TESSYS was applied to treat LSS favorably. Surgeons successfully decompressed the anterior nucleus pulposus under TESSYS with foraminoplasty but cannot remove posterior hypertrophic LF to achieve a fully dorsal decompression [13–15]. To counter this disadvantage, a modified TESSYS procedure—“U” route PELD—was developed recently in which the ventral articular joint was resected using a trephine under a visual endoscope to obtain enough exposure and the nucleus forceps was navigated ventrally to resect the anterior compressions after removing hypertrophied LF thoroughly, and this technique is catching more attention from spinal surgeons [16]. However, there is a paucity of data on investigating the superiority of “U” route PELD procedure on treating LSS combined with DH, and the conclusion is far from being confirmed.

To reach this question, patients diagnosed with LSS combined with DH who underwent “U” route PELD in our department were enrolled. The minimum dural sac cross-sectional area (mDCSA) was frequently measured to assess the severity of spinal canal stenosis [17], and the Visual Analog Scale (VAS) and Oswestry Disability Index (ODI) were regarded as valid and highly reliable instruments for pain estimation and disability measurement [18, 19]. The patients were all comprehensively evaluated at pre- and/or postoperation with VAS, ODI, and modified MacNab, and mDCSA at the surgical segment was measured using MRI. All patients were planned to be followed up for 2 years after the operation. Emerging evidence demonstrated that chronic noncancer pain is a biopsychosocial condition and is often accompanied by psychiatric and medical illnesses [20], including depressive symptoms and pain catastrophizing. The emotional disorders reciprocally aggravate pain and disability [21, 22]. Therefore, pain catastrophizing and depression were also assessed with the Pain Catastrophizing Scale (PCS) [23] and the Beck Depression Inventory (BDI) [24], respectively, for the patients at preoperation and postoperative 2-year follow-up. To further reveal emotional changes, including pain catastrophizing and depression between the LSS combined with DH patients who experience chronic pain and healthy individuals, we also collected questionnaires of PCS and BDI from 80 healthy individuals as controls whose age and sex matched with the patients.

## 2. Materials and Methods

### 2.1. Participants

This retrospective trial was approved by the Institutional Review Board of the Second Affiliated Hospital and Yuying Children's Hospital of Wenzhou Medical University. Protocols in detail were provided to each subject, and informed consent was obtained before the study. Eighty consecutive patients who received “U” route PELD during January 2014 and July 2018 were enrolled.

All included patients met the inclusion criteria: (1) patients with the diagnosis of degenerative LSS and combined DH (with or without lateral recess stenosis) on mono or double segments, with the imaging evidence of magnetic resonance images (MRI) and computed tomography (CT); (2) patients presented unilateral low back pain, limp, and sciatica that were consistent with the imaging evidence and have received conservative treatment more than 12 weeks but failed in symptoms relief; (3) patients agreed to accept “U” route PELD over other spinal surgeries; and (4) patients had lumbar MRI imaging in our hospital at pre- and postoperation. The exclusion criteria were (1) patients with spinal instability, including dynamic instability or more than grade II spondylolisthesis; (2) patients had a spinal surgical history; (3) patients with peripheral nerve disease, systematic infection, bleeding diathesis, or high risk of bleeding that cannot tolerate the surgery; (4) patients with mental illness and were uncooperative; and (5) patients lost to the follow-up.

### 2.2. Surgical Procedures

**Figure 1:**
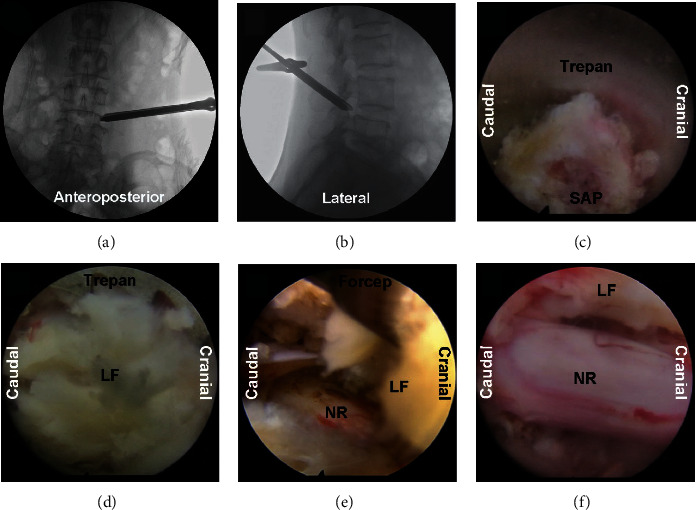
Intraoperative images of PELD of a case at L4-5 with “U” route PELD. The location of the working channel using fluoroscopy is showing in (a) anteroposterior and (b) sagittal. (c) The SAP was removed with a trepan for foraminoplasty. (d) The endoscopic image when trepan was introduced for posterior LF resection for dorsal decompression. (e) The forceps was introduced to remove dorsal LF. (f) Dorsal and ventral LF of the L5 nerve root was fully decompressed. SAP = superior articular process, LF = ligamentum flavum, and NR = nerve root.

All surgeries were performed by two senior and experienced surgeons, and all procedures were equipped with the transforaminal endoscopic spine system (Joimax GmbH, Karlsruhe, Germany). The following steps were performed in sequence in the “U” route procedures of PELD: (1) Patients who underwent the surgery were on the lateral position on an operating table on the contralateral side. The affected disc and pedicle were determined by the surgeon, who also designed the operation puncture length and angle under the guidance of C-arm fluoroscopy, and the skin entry was normally 10–12 cm from the midline. (2) After subcutaneously local anesthesia with 1.0–1.5 mL of 0.5% lidocaine, an 18-gauge puncture needle was inserted to the superior articular process (SAP) of the distal segment under fluoroscopic guidance. (3) The stylet was retreated, and then another 20 mL of 0.5% lidocaine was injected via the needle for further anesthesia, a guidewire passed through the needle, and then the needle was removed. (4) A skin incision about 0.8 cm diameter was made by a serial dilation with a cannulated obturator, and a working channel was rotated over the obturator. (5) The surgeon replaced the guidewire and obturator with a guide bar; a trepan was rotated along the working channel to SAP. Then, the position of the working channel was confirmed under the C-arm fluoroscopy. (6) An endoscope and two irrigation channels were inserted, and the ipsilateral-posterior hypertrophic and/or ossific LF were resected under endoscopic visualization after the foraminoplasty performance (Figure 1). (7) The working channel and endoscope were altered and navigated to the annulus to resect disc protrusion for ventral decompression until the neural root floated freely. (8) After full decompression and annuloplasty, the operation area was copiously irrigated and meticulous hemostasis was achieved.

### 2.3. Postoperative Radiography and Behavioral Measures

**Figure 2:**
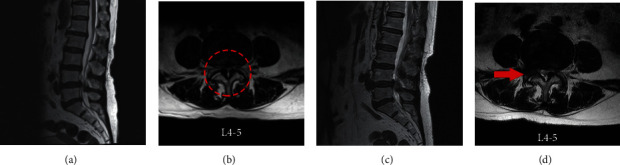
MRI scans of a case with lumbar stenosis at L4-5 with “U” route PELD. (a, b) Preoperative sagittal and cross-sectional MRI images, the lumbar stenosis, and disc herniation are indicated by the red circle. (c, d) Postoperative MRI images are showing ventral and dorsal decompression. The red arrow indicates that the removal of partly ventral osteophyte of SAP, herniated NP, and hypertrophic LF at L5. SAP = superior articular process, NP = nucleus pulposus, and LF = ligamentum flavum.

**Figure 3:**
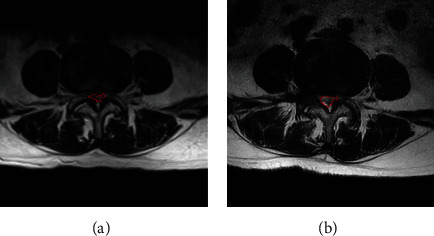
The cross-sectional area of the dural sac at the maximal compression level of a case with supine MRI scans. (a) Preoperative and (b) postoperative T2-weighted images plotted and measured on axial view using PACS. PACS = Picture Archiving and Communication System.

All patients were reexamined using a 1.5T MRI system (Signa HDxt; General Electric Company, Connecticut, USA) (Figure 2) and dynamic flexion-extension radiographs at postoperative 3-month. The radiological evaluation of mDCSA (mm^2^) at preoperation and were measured for all patients, the sagittal and axial T2-weighted images were obtained, and mDCSA at the most constricted lumbar spinal level of the surgical intervertebral disc was measured three times by the software of INFINITT Picture Archiving and Communication System (PACS) (INFINITT Healthcare Co., Seoul Korea) (Figure 3). The average was adopted for analysis [4]. All measurements were performed by two spine surgeons, who were mutually blinded and also to the patients' information. The difference was discussed between the 2 investigators until a consensus was reached.

A battery of self-reported questionnaires related to pain was filled before and after the operation to evaluate symptoms improvement. Low back and leg pain were estimated with VAS and the disability with ODI for the involved patients at preoperation, and postoperative 1-day, 1-week, 1-month, 3-month, 6-month, 1-year, and 2-year follow-up. The recovery condition was evaluated with modified MacNab criteria (excellent to poor) at postoperative 6-month, 1-year, and 2-year follow-up. Pain catastrophizing and depression of the patients were assessed with PCS and BDI at preoperation and postoperative 2-year follow-up, and the other 80 healthy individuals whose age and sex matched with the patients also completed PCS and BDI to detect the emotional changes of the patients. VAS is an 11-graded (0 to 10) visual scale to measure pain intensity, where 0 corresponds to no pain and 10 indicates the worst pain that almost cannot endure. ODI (0 to 100) assesses physical impairment related to pain. PCS (PCS total, PCS/t) includes three subscale that assesses aspects of helplessness (PCS/h), magnification (PCS/m), and rumination (PCS/r). BDI is a 21-item instrument for the severity of depression measurement.

### 2.4. Statistical Analysis

For continuous variables, data were presented as means ± standard deviations and were compared with Student's *t*-test or using Mann-Whitney *U* test if non-normally distributed, the Wilcoxon test was used for data analysis within groups, and the *post hoc* test was used for multiple comparisons. Qualitative data were presented as frequency and were analyzed using two-tailed chi-square tests or Fisher's exact test. Statistical analyses were performed using SPSS 25.0 software (SPSS Inc, Chicago, USA), and *p* value <0.05 was considered significant.

## 3. Results

### 3.1. Patients' Demographic Characteristics

Eighty patients receiving “U” route PELD were aged from 47 to 85 years old, with an average of 59.5 ± 9.76 years. The symptoms duration was 114.6 ± 22.77 months, and there were 2 cases operated at L2/3, 5 cases at L3/4, 39 cases at L4/5, 29 cases at L5/S1, 2 cases at L3/4 and L4/5, and the other 3 cases were at the levels of L4/5 and L5/S1. The operation time was 87.7 ± 25.20 minutes, and the average hospital stay was 5.8 ± 2.81 days (Table 1).

### 3.2. mDCSA and Pain Characteristics of LSS Combined with DH Patients

**Figure 4:**
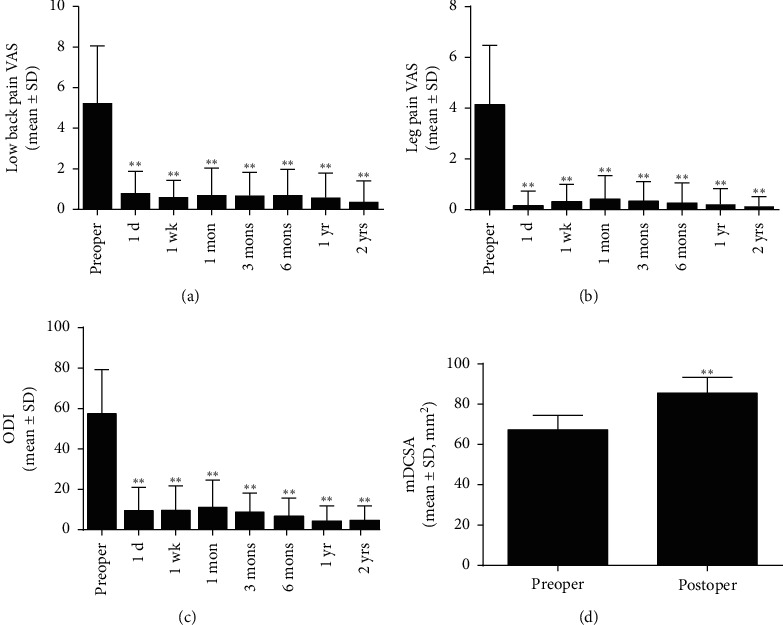
(a, b)VAS scores of the low back and leg pain, (c) ODI, and (d) mDCSA before and after the “U” route PELD surgery. VAS scores show the low back and leg pain rating and decrease at all postoperative time points compared with that preoperatively (*p* < 0.01). The postoperative mDCSA measured 3 months after surgery is significantly improved compared with preoperation. ^*∗*^*p* < 0.01, compared with preoperation.

As seen in Figures 3 and 4, the postoperative mDCSA in the patients was improved compared with that measured preoperatively (*p* < 0.001). The VAS of low back decreased significantly after the operation. It was reduced from 5.2 ± 2.82 to 0.8 ± 1.07 at postoperative 1-day follow-up, and to 0.4 ± 1.04 at the last follow-up (*p* < 0.001). The VAS of leg pain decreased from 4.1 ± 2.34 to 0.2 ± 0.57 at postoperative 1-day follow-up, and to 0.1 ± 0.38 at the last follow-up (*p* < 0.001). ODI decreased from 57.5 ± 21.76 to 9.48 ± 11.48 at postoperative 1-day follow-up, and to 4.7 ± 7.12 at the last visit (*p* < 0.001). The results showed a significant improvement in pain reduction and the physical function of the patients after surgical treatment.

**Figure 5:**
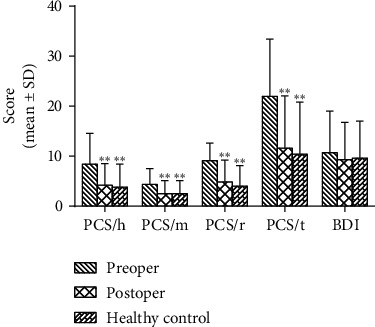
The scores of PCS (helplessness, magnification, rumination, and total) and BDI of the patients who underwent “U” route PELD and healthy individuals. PCS in the patients were evaluated at preoperation and postoperative 2-year follow-up, and the healthy individuals did not experience chronic pain and were age- and sex-matched with the patients. The postoperative PCS scores were all statistically decreased at postoperative 2-year follow-up compared with preoperation (PCS/h *p* < 0.001; PCS/m *p* < 0.001; PCS/r *p* < 0.001; PCS/t *p* < 0.001). The preoperative PCS score was significantly higher compared with healthy individuals (PCS/h *p* < 0.001; PCS/m *p* < 0.001; PCS/r *p* < 0.001; PCS/t *p* < 0.001), but no significance was found of PCS between patients at postoperation and healthy controls (PCS/h *p*=0.628; PCS/m *p*=0.923; PCS/r *p*=0.216; PCS/t *p*=0.462), and no significant difference of BDI between the patients and healthy individuals either at pre- or postoperation (preoperation *p*=0.388; postoperation *p*=0.787). ^*∗∗*^*p* < 0.01, compared with preoperation.

PCS and BDI were assessed for both patients and healthy individuals, as seen in Figure 5, and the scores of PCS were statistically reduced at postoperative 2-year follow-up compared with preoperation (Figure 5; PCS/h *p* < 0.001; PCS/m *p* < 0.001; PCS/r *p* < 0.001; PCS/t *p* < 0.001). Moreover, the preoperative PCS score was higher in LSS combined with DH patients than healthy individuals (PCS/h *p* < 0.001; PCS/m *p* < 0.001; PCS/r *p* < 0.001; PCS/t *p* < 0.001), but no significance of PCS between patients at postoperation and healthy controls (PCS/h *p*=0.628; PCS/m *p*=0.923; PCS/r *p*=0.216; PCS/t *p*=0.462) was found. However, we found no significant difference of BDI between the patients and healthy individuals either at pre- or postoperation (preoperation *p*=0.388; postoperation *p*=0.787).

### 3.3. Complications and MacNab Evaluation Outcomes

There were 2 patients out of touch at postoperative 1-year follow-up, and 2 patients died, so the remaining 76 patients completed the 2-year follow-up. The complications are shown in Table 1, and dural sac tear occurred in 1 patient, exiting nerve root injury in 1 patient, and 3 patients complained of dysesthesia after the operation. All of these patients recovered well after conservative treatment. There was 1 patient who needed a revision PELD surgery at the same segment within postoperative 1-year follow-up due to the unrelieved symptoms, and no recurrence, vascular injury, cauda equina injury, or infection was reported.

The patients have evaluated the postoperative recovery condition with modified MacNab criteria at postoperative 6-month, 1-year, and 2-year follow-ups, and the outcomes are shown in detail in Table 2. The excellent-to-good rate was 88.75%, 89.74%, and 93.42% at postoperative 6-month, 1-year, and 2-year follow-ups, respectively. The results indicated that patients recovered gradually after the operation, and the patients reached a better recovery condition at postoperative 2-year compared with postoperative 6-month and 1-year follow-ups (*p*=0.029).

## 4. Discussion

This retrospective study was designed to explore the therapeutic and safety of “U” route procedure of PELD for LSS combined with DH treatment. Most subjects involved in this study presented symptoms reliefs, with significant improvements in mDCSA on MRI. Despite the complications that occurred and 1 patient needed revision surgery, the “U” route PELD achieved identically feasible and safe for LSS combined with LDH therapy after comprehensive evaluations of VAS, ODI, and modified MacNab outcomes, and pain catastrophizing improvement was also observed.

Earlier studies have suggested that LSS patients should be decompressed under open surgery after the failure of a series of nonoperative therapies, but the open surgery remains challenging. It was reported the patients were evaluated a poor long-term outcome after decompressive laminectomy for LSS, 23% of patients received repeated surgery, and 33% of patients complained of severe back pain [25], and disadvantage factors of osteoporosis, advanced age, diabetes, and lumbar scoliosis also deteriorated the outcomes [26]. The surgeons always cut the articular joint tissue intraoperatively, but it has reached an agreement that excessive removal of facet joints is associated with spinal instability after open surgery [27]. To minimize the iatrogenic injury, the spinal surgeons were considering minimally invasive procedures for LSS treatment.

TESSYS PELD procedure, as a more minimally invasive technique than MED, was introduced to treat DH in the last decade. Due to the improvement of the PELD procedure and operative instrument, TESSYS was gradually indicated for LSS [13–15]. However, the traditional TESSYS PELD procedure was mainly indicated for ventral decompression and cannot achieve the resection of dorsal LF. A modified TESSYS procedure of the “U” route PELD that combined the ventral and dorsal decompression was then developed for lumbar stenosis with DH in recent years. Some study also reported the “U” route had been successfully used even for thoracic spinal stenosis with an excellent-to-good rate of 71.4% [28]. Foraminoplasty were performed in both procedures. To avoid spinal instability, less than 1/2 of ventral osteophyte on SAP was removed intraoperatively. The patients were all examined with dynamic flexion-extension radiographs to determine no spinal instability occurred (data not shown).

A published study reported postoperative 1-year clinical outcomes of 270-degree spinal canal decompression using TESSYS-ISEE technique for the patients with LSS combined with DH with a sample of 32 cases, a procedure similar to “U” route described in the present study [29]. Nevertheless, mDCSA was not analyzed, and emotional disorders were not evaluated in that previous study, so the exact extent of the spinal enlargement and the dynamic changes were unclear. In line to that study, we also observed that “U” route PELD significantly ameliorated the pain and life quality of the patients. We analyzed a sample of 80 patients diagnosed with LSS combined with DH with a longer follow-up duration of 2 years. The excellent-to-good rate ranged between 88.75% and 93.42% during postoperative 2 years, comparable with the previously published data [29].

mDCSA was frequently measured to assess the severity of spinal canal stenosis and was considered a more powerful variable in predicting the canal condition compared with diameters [16]. A smaller mDCSA predicted higher pressure on nerve roots [4, 17, 30, 31], and with a critical size of 75mm^2^; a further constriction would cause increased pressure on the nerve root [27]. The preoperative average of mDCSA was 67.3 mm^2^, which is lower than the reported critical size. We found mDCSA at the surgical level was statistically enlarged by the “U” route PELD. Still, there is a limitation that the mDCSA was measured at postoperative 3-month follow-up. The long-term postoperative changes of mDCSA were not detected in this study.

Besides, the mDCSA was a strong predictor of clinical symptoms and quality of life of LSS patients [4, 17]. Although previous studies have demonstrated that mDCSA of MRI changed significantly with the position, symptoms would be aggravated with worsened dural sac constriction from supine to standing, and we also found a significant improvement in mDCSA between pre- and postoperation with supine MRI examination. Our results indicated that mDCSA on supine predicted clinical symptoms and life quality, although the correlation was reported moderate to weak [31].

A biopsychosocial model of chronic pain has been widely adopted, and epidemiologic studies suggested that chronic pain increases the risk for depression, anxiety, and pain catastrophizing [20, 21]. The coexistence of chronic pain and emotional disorders initiates vicious pain, which spirals and amplifies depression and pain catastrophizing and then disturbs daily activities and consequently further exacerbates pain [32–34]. Depression is a leading cause of disability globally. The World Health Organization estimated that about 350 million people suffer from depression worldwide [35]. Catastrophizing is defined as a specific psychosocial construct comprising negative cognition and emotional processes dealing with physical and psychical pain, including helplessness, magnification, and rumination [36]. Due to the characteristics of internal and private experience of pain, self-report is considered to be the gold standard for its measurement [37]. The authors evaluated the patients in this study with self-reported questionnaires, including PCS and BDI, in addition to VAS, ODI, and MacNab. Pain catastrophizing significantly decreased in patients after “U” route PELD treatment, and the difference was not significant between the patients at postoperation and healthy controls. Besides, no significance of BDI between patients and healthy individuals was found either at pre- or postoperation. The average of BDI in patients or healthy individuals all among 9 to 11 suggested that both patients and healthy controls had moderate depression, which may be attributed to the pain duration and advanced age. Depression is reported highly prevalent in elderly population, and pain has been demonstrated by sharing common biological pathways and neurotransmitter mechanisms [38, 39]. Human functional magnetic resonance imaging (fMRI) studies have shown persistent pain leads to significant changes in the brain, such as greater functional connectivity between the nucleus accumbens and prefrontal cortex, decreased brain gray matter density, and less hippocampus volume [40, 41]. The neuromechanisms of BDI may be changed as well by chronic pain; therefore, BDI in the patients did not change 2 years after operation even though VAS and ODI have been significantly improved. According to the results, the healthy controls suffered moderate depression, but without chronic pain, which may be related to stress in life and loss of physical, sensory, and other resources.

The occurrence of complications of nerve root injury, dysesthesia, and dural sac tear was inevitable in the “U” route PELD. The foraminoplasty and decompression were all performed under endoscopic visualization, so the mechanical tear and nerve root injury resulted from surgical tool compression could be avoided. The nerve root injury and dysesthesia might be caused by thermal injury when using high-speed endoscopic burr and side-firing laser in the process of undercutting of SAP [42]. The patients with nerve root injury or who complained of dysesthesia were cured conservatively, and all recovered within 3 months after operation. Dural sac tear mainly occurred when resecting the nucleus pulposus or LF that tightly adhere to the dural sac. The dural sac tears in this study were found intraoperatively. Then, a small piece of gelatin-sponge was applied for sealing under the endoscope. All the patients were symptoms free after the operation. It is worth noting that one patient needed a revision PELD surgery at the same segment due to unrelieved symptoms. All the reoperations happened within postoperative one year. The outcomes suggested a possibility of insufficient decompressions of “U” route procedure of PELD, which is consistent with a previous study [29].

The novelty of the present study was exploring the therapeutic effect of the “U” route PELD on LSS with DH and detected the improvement of emotion in these chronic pain patients. Despite being a preliminary study, “U” route PELD seems promising with a superior clinical outcome within postoperative 2 years. The posterior interlaminar approach is also indicated for central spinal stenosis but cannot completely remove the ventral disc protrusion. Therefore, the posterior interlaminar approach was not included in the comparison in this study. The investigation of the superiority of the posterior interlaminar approach of PELD for central LSS treatment without DH would be carried out shortly.

In conclusion, “U” route procedure of PELD seems a feasible and safe PELD procedure for LSS combined with DH treatment, which achieves significant improvements in mDCSA, clinical symptoms, and pain catastrophizing. However, the risks of complications and inadequate decompression are potential. This is a retrospective study with preliminary results, and a more confirmative conclusion needs randomized trials with a larger sample size in the future.

## Figures and Tables

**Table 1:** The demographics and complications of the patients and healthy controls.

	“U” route PELD (*n* = 80)		Healthy controls (*n* = 80)		*p* value
Age, mean, SD (y/o)	59.5	9.76	56.7	8.10	0.078
Male, *n* (%)	49	61.25	47	58.75	0.821
Symptom duration, mean, SD (month)	114.6	22.77	na	na	na
Operation time, mean, SD (minute)	87.7	25.2	na	na	na
Hospital stay, mean, SD (day)	5.8	2.81	na	na	na
Lost to follow up, *n* (%)	4	5	na	na	na
Revision operation, *n* (%)	1	1.25	na	na	na
Dural tear, *n* (%)	1	1.25	na	na	na
Nerve root injury, *n* (%)	1	1.25	na	na	na
Dysesthesia, *n* (%)	3	3.75	na	na	na

**Table 2:** The outcomes of modified MacNab evaluation at postoperation.

Outcomes	Excellent	Good	Fair	Poor	*p* value
6-month, *n* (%)	45 (56.25)	26 (32.5)	7 (8.75)	2 (2.5)	0.029
1-year, *n* (%)	60 (76.92)	10 (12.82)	7 (8.98)	1 (1.28)	
2-year, *n* (%)	56 (73.68)	15 (19.74)	4 (5.26)	1 (1.32)	

## Data Availability

The data used to support the findings of this study are available from the corresponding author upon request.
